# Flavor Scalping in Packaged Foods: A Review

**DOI:** 10.3390/molecules31081358

**Published:** 2026-04-21

**Authors:** Michael G. Kontominas

**Affiliations:** Laboratory of Food Chemistry and Technology, Department of Chemistry, University of Ioannina, 45110 Ioannina, Greece; mkontomi@uoi.gr

**Keywords:** flavor compounds, absorption, polymers, factors affecting absorption, beverages, foods

## Abstract

Over the past decades, plastics have been increasingly employed to package foods and beverages. Furthermore, foods, nowadays, are kept in contact with plastics for far longer periods than ever before. A number of conventional polymers, i.e., polyethylene (PE), Polypropylene (PP), Ethylene Vinyl Acetate (EVA), Εthylene vinyl alcohol (EVOH) polystyrene (PS), Polyvinyl chloride (PVC), Polyvinylidene chloride (PVDC), polyethylene terephthalate (PET), Polycarbonate (PC), polyethylene naphthalate (PEN), Polyamides (PAs), Polyacrylonitrile (PAN) as well as biodegradable polymers-[Polylactide (PLA)] are used commercially in food packaging applications. Potential interaction of food with the packaging container includes: permeation, migration and flavor scalping. Most food and beverage containers are lined with plastics mainly polyolefins, which due to their low polarity tend to absorb volatile compounds of similar polarity. Absorption of flavor compounds by polymers involves both partitioning and diffusion through the plastic. Absorption is influenced by (i) polymer properties such as polarity, morphology, glass transition temperature, density, free volume, crystallinity and surface area, (ii) flavor compound properties such as structure, concentration, and polarity, and (iii) external factors such as temperature, time of contact, relative humidity and the proximity of other compounds. Based on the above, it is apparent that flavor scalping should be among one of the food packaging industry priorities in order to efficiently preserve the quality of packaged food flavor. This review highlights the various factors affecting the scalping process, as well as the consequences of flavor scalping in various food and beverage commodities. The review covers the period 1990–2925 and used the LitChemPlast data base for literature search.

## 1. Introduction

Among the various functions of a food packaging, the protection and preservation of the contained product quality and safety are by far the most important [1]. According to the food packaging industry, approximately 70% of all foods and beverages come into intimate contact with plastics at some point in time during their production and distribution cycle. The replacement of conventional packaging materials such as metal, glass and paper by plastics is justified by the many advantages of the latter including: low cost, ease of processing to form containers of various shapes and sizes, light weight, shatter resistance, transparency (in most cases), flexibility, good mechanical properties, etc. Disadvantages that limit the use of plastics in food and beverage packaging include: low heat resistance (many foods require pasteurization or sterilization), high gas and water permeability (for numerous plastics), and interaction of the packaging material with the contained food/beverage in the form of (a) migration of low molecular weight compounds and (b) absorption of food/beverage flavor (flavor scalping) [1,2,3].

**Figure 1:**
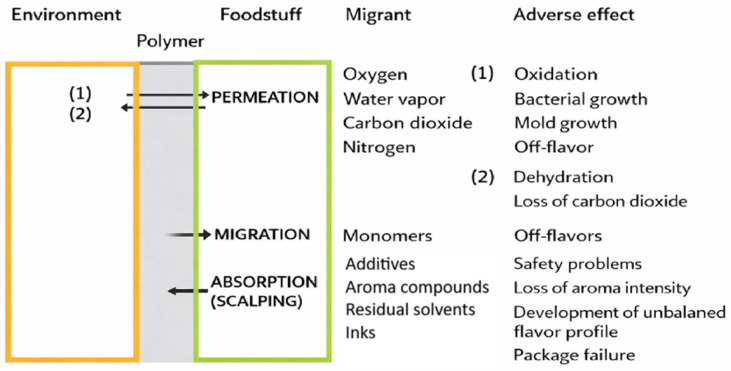
Mass transfer between environment/package/food.

Mass transfer between environment/package/food product includes: **(A) Permeation** involves the diffusion of fixed gases or organic vapors through homogeneous packaging materials, excluding the passage through perforations, cracks, or other defects. Permeation occurs through two basic mechanisms: (i) diffusion of molecules across the package wall (driven by a concentration gradient), and (ii) absorption/desorption of molecules from/into the internal/external atmospheres (Figure 1). Permeation significantly affects the shelf life of packaged foods, as permeating molecules such as oxygen or water vapors cause undesirable chemical reactions or physical changes in the packaged product resulting in the deterioration of product sensory properties, quality and even safety [1,3,4,5].

**(B) Migration** is the transfer of low molecular weight (MW) compounds present in the polymeric packaging material (e.g., monomers, oligomers, plasticizers, UV-stabilizers, antioxidants, printing inks, solvents used in adhesives, etc.) into the packaged food. Migration involves the **diffusion** of migrants from the packaging material matrix to the packaging material/food interface, controlled by Fick’s law and **desorption** of the migrants into mass of the food, controlled by Henry’s law. As with permeation, the process of diffusion is driven by a concentration gradient. As above-low MW compounds are not chemically bound to the polymer macromolecular chain, they are able to diffuse through the polymer matrix. Migration may result in undesirable changes in the packaged food including sensory and nutritional changes and even in safety issues [1,3,4,5].

**(C) Flavor absorption (flavor scalping)** is the undesirable absorption of flavor volatile compounds from food into the packaging material, leading to a loss of food flavor quality, or conversely, the absorption of undesirable flavors from the packaging into the food, degrading the product’s sensory properties [6]. Τhe characteristic flavor of a particular food is usually the result of hundreds of individual volatile compounds interacting to produce a recognizable aroma and taste. In other cases, flavor is the result of a single compound (i.e., vanillin in vanilla). Thus, if one or more flavor compounds are altered or lost, the sensory food quality may be compromised. Typical examples of flavor scalping are (i) the absorption of limonene, the major flavor compound of orange juice, by the polymeric packaging material and (ii) the uptake of ethyl acetate by extruded puffed snacks packaged in multilayer barrier films. In this case, ethyl acetate is the solvent in the adhesive material used to prepare the multilayer film structure. Flavor compounds (<400 Da) are low MW compounds, usually nonpolar, not only readily diffusing through plastics but also soluble mostly in nonpolar polymers. The result of the absorption of aroma compounds by plastic packaging materials is the creation of an unbalanced food flavor profile [3,4,7]. Furthermore, the absorbed food molecules can affect the packaging integrity properties (barrier and/or mechanical properties) [3,4]. Considering that the most prominent function of an efficient food packaging material is protection, mass transfer into or from the packaging material should be minimized. Food industries often deal with the flavor scalping problem by adding excess flavors to the food in order to retain acceptable product flavor until the end of the food’s shelf life [8]. The major factors affecting the phenomenon of flavor scalping include: (i) flavor compound properties, (ii) polymer properties and (iii) external factors.

## 2. Flavor Compound Properties

### 2.1. Concentration

According to Fick’s law, the higher the initial concentration of the sorbed flavor compound, the higher the rate of transport into the polymer structure and, consequently, the higher the equilibrium amount of sorbed compound in the polymer matrix. For example, Farhoodi et al. [9] showed that the higher the concentration of limonene in citrus beverages, the higher the absorption of flavor by PET bottles. In the case where a co-permeant is present, the rate of transfer usually increases [7,10]. Furthermore, as the concentration of flavor compounds increases, their sorption into a polymer material may cause swelling of the polymeric matrix. Swelling, in turn, of certain polymers by some flavor compounds may result in increased oxygen transmission through the packaging material which may affect food quality, i.e., through oxidation. Absorption of flavor compounds may also cause delamination of multilayer packages [7]. As both solubility and diffusivity of the migrant molecule are directly related to its concentration, diffusion coefficients increase with increasing permeant concentration [11].

### 2.2. Molecular Weight, Carbon Chain Length, and Structure

**Figure 2:**
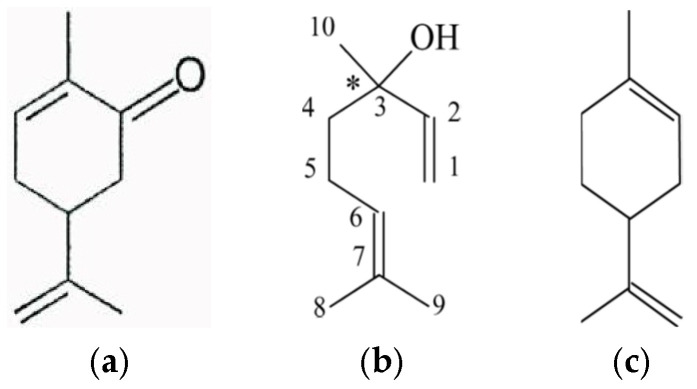
Structures of (**a**) linalool, (**b**) limonene and (**c**) carvone. (* = assymetric carbon atom).

The carbon chain length of a molecule is closely related to its boiling point and its solubility. Increased MW of the migrant molecule results in greater solubility. In a study by Linssen et al. [12], it was shown that (i) compounds with eight or more carbon atoms were easily sorbed by high density polyethylene (HDPE) from yogurt drinks, with shorter molecules remaining in the product, and (ii) highly branched molecules were sorbed to a lower extent than linear molecules. Charara et al. [13] correlated the absorption of aldehydes to their structure, with the shorter chain aldehyde, C_10_ (decanal), being absorbed to a lesser extent than the C_12_ (dodecanal). Strandburg et al. [14] explained the differences among esters with respect to their absorption potential: the longer the ester molecule chain, the lower its polarity and the higher the absorption of the compound by the nonpolar polyolefins. Ronald and Hotchkiss [15] compared the solubility of limonene to that of linalool in PE and justified the higher solubility of the former based on the fact that (i) linalool is a linear, less bulky molecule than limonene, which facilitates its ability to penetrate more easily into a given polymer matrix, and (ii) the higher b.p. of linalool (198 °C) compared to that of limonene (175 °C) is indicative of its ability to condense and remain within the polymer matrix (Figure 2).

Arora et al. [16] conducted a study on the sorption of esters, aldehydes and sulfur compounds in a milk simulant by LDPE. Sorption increased with increased carbon chain length for esters, aldehydes and ketones. The sorption was 11–63% for aldehydes (C_7_–C_10_); 1.5–43.0% for methyl ketones (C_7_–C_10_); 6.0–42.0% for methyl esters (C_7_–C_9_); and 8.5–21% for sulfur compounds. The shorter chain C_6_ aldehyde (hexanal) was absorbed less than the C_10_ aldehyde (decanal). Similarly, van Willige et al. [2] showed that with increasing carbon chain length, the molecule polarity decreases, resulting in an increase in absorption. Finally, Farhoodi et al. [9] reported the absorption amount of flavors (d-limonene, a-pinene and myrcene) in cola, orange and lemon beverages by PET bottles to be dependent on the structure and initial concentration of the flavor compounds, type of surrounding medium and storage temperature. Limonene was the only flavor that was absorbed into the PET bottles from all three beverages.

### 2.3. Polarity and Functional Group

Flavor compound polarity comprises a major controlling factor affecting sorption. Flavor compounds are absorbed more easily in a polymer of similar polarity according to the principle ’like dissolves like’. Lipophilic compounds will be preferentially absorbed by lipophilic polymers. Lipophilicity increases with MW within a homologous series. Work has shown that the sorption of a number of citrus flavor compounds by LDPE depends on the polarity of the compounds. Between carvone (C_10_H_14_O) and limonene (C_10_H_16_), both terpenes, it has been shown that the less polar limonene is sorbed at a faster rate by the nonpolar LDPE [16]. Terpenes showed the highest affinity for the polyolefins, followed by sesquiterpenes (C_15_). With respect to other flavor compound classes, esters and aldehydes sorbed at a higher rate compared to alcohols, due to lower polarity [17]. Likewise, polar volatile compounds such as benzaldehyde, citral, and linalool, are absorbed much less by PE compared to limonene or other nonpolar hydrocarbon-based volatiles. Work on sorption of orange juice flavor compounds by LDPE demonstrated that hydrocarbon compounds showed the highest affinity to the polymer, followed by ketones, esters, aldehydes, and finally alcohols [12,18,19].

Other important factors affecting sorption include the molecular size of the flavor compounds as well as the solubility properties of both the polymer and the flavor compounds. In general, the smaller the difference in δ values (Hildebrand solubility parameter) of the polymer and the aroma compound, the higher the solubility of the flavor compound will be in the polymeric matrix, i.e., alcohols (polar compounds) have a much larger δ solubility parameter values than polyolefins, and thus, will be less absorbed compared to aldehydes and esters. Esters and aldehydes have solubility parameter values very close to LDPE, linear low density polyethylene (LLDPE), and PP, which partly explains the large extent of their sorption by these polymers. Regarding the varying extent of sorption shown for esters, this can be accounted for by differences in the length of the carbon chain of the ester molecule. That is, the longer the chain, the less polar the compounds, resulting in higher absorption by the nonpolar polyolefins [20]. However, Peychès-Bach et al. [21] reported that the sorption of flavor compounds in PE film could not easily be predicted since besides polarity and chemical nature of the aroma compound, other parameters such as competition between aroma compounds and/or interactions with the polymeric matrix should also be considered. Along the same line of reasoning, Licciardello et al. [22] reported that the difference in polarity between ethyl octanoate and linalool justified the higher sorption potential of the former for LLDPE and cast polypropylene (CPP) film.

Molecules with a linear structure sorb more easily into the polymer matrix compared to cyclic/aromatic molecules due to their smaller molar volume. Also, aliphatic compounds sorb more easily compared to aromatic/cyclic compounds of similar MW. Branching or unsaturation in the backbone of the molecule (for an equal carbon number) causes a decrease in sorption as shown for 3-methyl butyl-3-methyl butanoate compared to hexyl butanoate in PET due to a steric hindrance effect. For compounds with a linear structure and comparable mass, the rate of sorption varies according to the following order: ester > ketone > aldehyde. The maximum sorption was observed for flavor compounds possessing a long chain length, i.e., the penetration behavior of dodecyl acetate appeared higher than that of hexyl acetate [23].

The structure and functional groups of solutes exert a strong influence on sorption behavior towards polymers of similar polarities. The hydrocarbon solutes limonene, myrcene and pinene were sorbed significantly more than the more polar alcohol, aldehyde and ketone solutes (citral a and b, carvone) by polyolefins of different branched structure but similar polarities [24].

## 3. Polymer Characteristics

### 3.1. Modeling of Flavor Scalping

Unidimensional mass transfer, in this case the absorption/desorption of flavor compounds by/from a polymer, is described by Fick’s second law [7]:

(1) $$\frac{dc}{dt}=\frac{d}{dx}(D\frac{dc}{dt})$$

where *c* is the concentration of the absorbed molecule, D is the diffusion coefficient of the absorbed molecule, *t* is the time for diffusion and *x* is the distance covered by the absorbed molecule.

The analytical solution of Equation (1) is given by Equation (2):

(2) $$M(t)=M_{\infty }\left\{1-\frac{8}{\pi^{2}}\sum_{n=0}^{n=\infty }\frac{1}{{\left(2n+1\right)}^{2}}\exp\;[-D{\left(2n+1\right)}^{2}\pi^{2}\frac{t}{4L^{2}}]\right\}$$

where *M*(*t*) (μg cm^−2^) is the amount of absorbed molecule by the polymer at time (*t*), *M*∞ (μg cm^−2^) is the amount of absorbed molecule at equilibrium, *D* (m^2^ day^−2^) is the diffusion coefficient of the absorbed molecule, *L* (μm) is the thickness of the polymer and *t* (m) is the thickness of the polymer.

Equation (3) can be applied to describe, approximately, the migration of a substance from a polymer into a food (simulant) for situations where *m_F_*_,*t*_/*m_F_*_,*e*_ < 0.5:

*m_F_*_,*t*_ = 2 *k c_p_*,_0_ (*D_p_ t*/*π*)^1/2^ (3)

where *m*_*F*,*t*_ is the amount of migrant into the food phase at time *t*, *m_F_*_,*e*_ is the amount of migrant at equilibrium, *c*_*p*,0_ is the initial concentration of migrant in the polymer matrix, *Dp* is the migrant diffusion coefficient, and constant *k* is the measure of the influence of factors that lie outside the polymer/migrant system and has, by definition, a value of *k* = 1 in the absence of such influences (such as for contact with ethanol and oil) as long as no polymer swelling takes place.

The diffusion coefficient (*D*) of the flavor compound is a very important parameter in order to predict the flavor quality of the packaged food. The diffusion coefficient has a direct relationship with temperature. The dependency of the diffusion coefficient on temperature is given by the Arrhenius equation:

(4) $${D=D_{0}\;e}^{Ea/RT}$$

where *D*_0_ is the hypothetical diffusion coefficient at very high temperature, *E*a (Jmol^−1^) is the activation energy of diffusion, *R* (J mol^−1^ K^−1^ is the gas constant, and *T*(K) = 273 + *t* (°C) is the temperature. *D*_0_ and *E*a can only be obtained by fitting with experimental data.

Besides diffusion, mass transfer from a food into a polymer also involves equilibrium partitioning between the package and food. The most important variables that control mass transfer include: the partition coefficient, time and temperature. The partition coefficient (*k_P_*) is defined as the concentration ratio at equilibrium of the volatile compound in the polymer (*C_P_*) divided by that at equilibrium in the foodstuff (*C_F_*) or food (Equation (5)). The values for *k_P_*, range over several orders of magnitude, depending on the polarity of the polymer involved, the type of food and the nature of the flavor compound.

*k_P_* = *C_P_*/*C_F_* (5)

Finally, a measure of the compatibility between the polymer and the flavor compound, the solubility coefficient (*S*) influences partition coefficients, thus giving it the potential to predict the scalping potential of the packaging material. The solubility of an organic vapor is defined as:

*S* = *M*_∞_/*ν* × *p* (6)

where *M*_∞_ is the total mass of vapor absorbed by the polymer, *p* is the driving force ex pressed in Pascal, and *ν* is the film volume. The unit of *S* is (kg/m^3^.P).

Based on the above, the permeability coefficient (*P*) can be defined as the product of the diffusion coefficient (*D*) and the solubility coefficient (*S*).

*P* = *D* × *S* (7)

To illustrate the significance of the partition coefficient in flavor scalping, the following example is given. The partition coefficient of d-limonene, the major flavor compound in orange juice, between PET and orange juice generally ranges from approximately 2 × 10^−5^ to 2 × 10^−3^ indicating a relatively low affinity of PET for limonene compared to LDPE for which respective partition coefficient ranges from 1 × 10^3^ to 3.4 × 10^3^. This indicates that the concentration of d-limonene in the LDPE at equilibrium is over 3000 times higher than its concentration in the orange juice. Limonene is rapidly absorbed by LDPE from orange juice, with equilibrium often reached within 2 to 3 days, reducing the concentration of d-limonene in the juice by up to 50%. The respective absorption of d-limonene by PET, including in refillable PET bottles, does not exceed 2–3% of the total d-limonene in the product.

### 3.2. Surface Area and Thickness

The extent of flavor compound sorption at equilibrium is directly proportional to available surface of the packaging container with the food. The larger the contact surface area between the food and the polymer, the greater aroma will diffuse into the polymer. Furthermore, plastics containers of different thickness may result in different sorptive behavior [22] with extent of sorption increasing with increasing polymer thickness. In case of multilayer packaging materials with an inner polyolefinic layer in contact with the food, it was shown that the time required for equilibrium conditions to be reached is shorter due to the low thickness of the polyolefinic layer [22].

### 3.3. Polarity

With regard to the sorption potential of flavor compounds, these are absorbed more easily in polymers of similar polarity [25]. Polyolefins are nonpolar polymers and have a high affinity for nonpolar molecules (nonpolar flavor compounds, fats, oils), these showing high sorption potential by the polymeric package [26]. However, polymers such as polyesters (PET) are more polar than polyolefins and thus exhibit a lower sorption potential for the nonpolar compounds; for example, the solubility of polar ketones in PET is approximately two times higher than that of nonpolar alkanes having the same chain length and a similar carbon skeleton [22].

Table 1 shows the approximate partition coefficient (*kp*), values encountered in plastic/food systems based on the polarities of the solutes, plastics, and foods [27]. Polar aroma components are sorbed to a greater extent into polymers in the presence of a nonpolar fatty food simulant, and vice versa. Nonpolar compounds are sorbed less in the polymer than polar ones in the presence of n-heptane, iso-octane and sunflower oil. As aroma polarity increases, they are retained by PET to a greater extent [26].

van Willige et al. [28] investigated the sorption of various flavor compounds (limonene, myrcene, decanal, hexyl acetate, nonanone, carvone, linalool, octanol, and hexanal) by oriented polypropylene (OPP) and LLDPE. Both nonpolar polymers easily absorbed the nonpolar hydrocarbons (limonene and myrcene) showing less affinity for the moderately polar decanal, hexyl acetate, nonanone, carvone, linalool, octanol, and hexanal. Among the above flavor molecules, even though carvone and limonene have similar structure, limonene is a nonpolar terpene, while carvone is an oxygenated polar terpene (Figure 2). Thus, limonene was more easily absorbed by polyolefins compared to carvone. On the other hand, PET, a polymer of relatively higher polarity, absorbed lower amounts of seven terpenes of different molecular structures and functional groups. In a similar study, Licciardello et al. [22] reported that the aroma compounds ethyl octanoate and linalool were more easily absorbed by CPP compared to LLDPE suggesting that the nature of the aroma compound can be more important with regard to the extent of sorption than the effect of the plastics material in determining the differences observed. This is not surprising given the similarity in chemical structure of the two plastics tested, both being polyolefins.

The diffusion coefficient of ethyl octanoate in LLDPE is about 13-fold lower than the linalool diffusion coefficient; whereas, the difference is only 1.2-fold for the investigated volatiles absorbed into CPP [22]. Limonene was sorbed more by LDPE than by HDPE (61 vs. 43%) and PP sorbed limonene only slightly more than LDPE (64% and 69% vs. 61%) but more than HDPE (43%). Thus, the order of affinity of limonene sorption was PP ≈ LDPE > HDPE. The solute polarity was the predominant controlling factor and polymers with similar low polarities had a lesser effect on sorption [2].

### 3.4. Glass Transition Temperature

The glass transition temperature Tg can be defined as the temperature at which polymer molecular chains start to move when subjected to an external force. The rate of sorption of flavor compounds is dependent, to a certain extent, on the glass transition temperature (Tg) of the polymer which, in turn, is a measure of the flexibility of the polymer macromolecules. Polymer molecules, below Tg, are stiff corresponding to the glassy state of the polymer and the possibility of a flavor molecule to penetrate the polymer matrix is limited due to a dense structure having little void volume. Above Tg, the polymer molecules are highly flexible corresponding to the rubbery state, which allows flavor compounds to enter the polymer matrix. Rubbery polymers such as the polyolefins PE and PP, have a Tg below room temperature, and a high diffusion coefficient for flavor compounds with sorption equilibrium being established quickly in such structures [29]. Polymers such as PET, PC, and PEN have a Tg above ambient temperature. These glassy polymers have very stiff chains at room temperature and, thus, very low diffusion coefficients for flavor molecules at a low concentration. Likewise, glassy polymers such as PVDC have a low diffusion coefficient for flavor molecules at a low concentration and hence display excellent flavor-barrier properties [10]. In comparison to PET, PEN has excellent barrier performance for the packaging of carbonated beverages, allowing hot-fill, rewash and reuse, due to its high Tg [30].

It is well accepted that solvent molecules/flavor compounds within the polymer matrix function as a plasticizer, resulting in increased mobility of the polymer chains, consequently lowering polymer Tg. Thus, an initially glassy polymer may exhibit a rubbery character as a result of an increase in solvent concentration (absorption) [29,31]. Leelaphiwat et al. [32] determined the diffusion and permeability coefficients of selected flavor compounds (eucalyptol and estragol) in contact with LDPE, PP, nylon, PET, MPET and PLA films. Results showed that (i) diffusion coefficients were the highest in LDPE for estragol, (ii) permeability coefficients were the highest in PLA for estragol, (iii) permeability coefficients were the highest in PP and LDPE for eucalyptol and (iv) permeability coefficients were the lowest in metPET for both flavor compounds studied. Van Willige et al. [2] investigated the absorption of flavor compounds by LLDPE, oriented polypropylene (OPP), PC, PET, and PEN. Results showed that (i) flavor absorption by LLDPE and OPP was 3 to 2400 times higher than by PC, PET, and PEN. These differences were justified by differences in Tg of the materials, (ii) the polyolefins (LLDPE and OPP) being in the rubbery state at the temperature tested, easily absorbed limonene and myrcene followed by decanal, hexyl acetate, and nonanone, (iii) despite the fact that the Tg of PC was much higher than the Tg of PET and PEN, absorption of flavor compounds by PC was much higher than by PET and PEN. This behavior was attributed to the very low of crystallinity in PC, which is a totally amorphous polymer, (iv) given the more polar character of PC, PET, and PEN, the nonpolar terpenes limonene and myrcene showed lower absorption than the rest of the abovementioned flavor compounds. Due to the very high Tg’s of PC, PET and PEN these polymers were in the glassy state resulting in very low diffusion coefficients for flavor compounds and thus, functioning as flavor-barrier materials [29] and (v) differences in Tg may also explain differences in amounts of absorbed flavor compounds between PET and PEN [2].

### 3.5. Crystallinity and Orientation

Crystallinity refers to the degree of close packing of macromolecular chains within the polymer matrix. Most polymers exist as complex structures made up of crystalline and amorphous regions. Crystallinity is usually induced by heating above the Tg [33]. Orientation refers to the degree of preferential alignment of the macromolecular polymer chains. Polymer crystallinity and orientation leading to three-dimensional order of the polymer structure influence permeability and diffusivity [10]. The more ordered the polymer molecular structure, the lower the rate of sorption of food flavor compounds. Crystalline regions can, also, act as crosslinks to prevent swelling of the polymer. Furthermore, crystalline regions can also affect solute permeability by restraining polymer segmental mobility in the amorphous regions. Thus, the degree of sorption and diffusion for a given solute is directly related to the degree of the amorphous polymer content [13]. For example, increase in crystallinity of PE and nylon 6 reduces diffusivity of oxygen up to five times [4]. Estimated diffusion coefficients for amorphous polyethylene terephthalate (APET) are one order of magnitude higher than those for branched polyethylene terephthalate (BPET) due to the higher degree of crystallinity and orientation in BPET. Diffusivity is also affected by orientation in glassy polymers such as PET [33].

### 3.6. Polymer Density

The density is an indication of free volume in the polymer matrix. The higher the polymer density, the lower the free volume and the lower the permeability and solubility of the flavor compounds in the polymer [4,34]. The rate of sorption for limonene decreased from 75% to 63% and that of carvone from 17% to 10% when the PP density increased from 0.883 g/cm^3^ to 0.921 g/cm^3^, respectively [4].

### 3.7. Processing History

Studies have shown that the sample processing history may affect morphology of both the crystalline and non-crystalline regions. Such morphological differences can be reflected in the extent of sorption [35]. Polymer morphology changes during the forming process are owed to rearrangement of the crystalline and amorphous regions. Such rearrangements may affect solute sorption rates by affecting polymer characteristics such as polarity, Tg, free volume and crystallinity [36,37].

### 3.8. Use of Recycled Plastics

Within the general trend for more efficient municipal waste management including recycling of plastics, refillable PET beverage bottles have been adopted and used in many countries. A major problem associated with refilling procedure is the sorption of flavors from foodstuffs by the PET material, responsible for the development of off-flavors in the products filled into the same container. The extent of sorption is affected by a number of factors including polymer type, carbon chain length, type of functional groups of the sorbate and temperature. Gremli [35] reported that washing of used plastic bottles with NaOH solutions removed less than 50% of the sorbed amount of terpenes. Safa et al. [38] investigated the efficacy of washing of used PET bottles with NaOH solution in order to remove the sorbed flavor compounds: limonene, linalool, and linalyl acetate. Results showed that even after washing limonene remained substantially sorbed into the bottle walls. Furthermore, recycled material may be used in new multilayer bottles provided that a virgin polymer layer is placed between the recycled polymer and the food this acting as a functional barrier [39,40].

## 4. External Factors

### 4.1. pH

According to published studies, pH may or may not affect sorption of flavor compounds by polymeric packaging materials depending on the sorbent and the plastic involved. pH has been shown to affect sorption by a factor of 40 between pH 3 and 5 for 2-hexanal into PE [41]. Furthermore, the same authors, regarding the interaction of flavor compounds such as *trans*-2-hexanal, 2-heptanone, 6-methyl-5-hepten-2-one, 6-methyl-5-hepten-2-ol, and limonene from tomato juice with polymers such as PET, PE, and EVOH as a function of pH, reported that the amount of extractable flavor compounds in the different polymers at all pH values decreased in the following order: EVOH > PE > PET. Among these polymers, the sorption of the flavor compounds was less affected by pH in PET compared to the other two polymers. PE sorbed larger amounts of the compounds at pH 4 than at pH 7. Regarding EVOH, an increase in sorption of *d*-limonene was shown with increasing pH. It was rationalized that pH directly affects polarity, solubility, and structure of flavor molecules. Likewise, Caner et al. [42] reported that the pH of the food simulant affected the sorption of aroma compounds into the packaging material. That is, lower amounts of d-limonene were sorbed in the presence of 3% acetic acid compared to 10% ethanol for all structures (PP, PE/PA/EVOH/PE; and metPET/EVA/LLDPE). Sorption of limonene was shown to be 1.3 times higher in LDPE at 22 °C at pH 5.2 than at pH 2.6 [43]. On the other hand, a study on limonene in orange juice showed pH to have no significant effect on flavor scalping [18]. Likewise, Matsui et al. [44] reported that the pH of flavor solutions had no effect on the sorption of flavor compounds into PE film. Similarly, Nielsen & Jägerdsad, [45] reported no significant effect of pH on the sorption of flavor compounds.

### 4.2. Temperature

**Figure 3:**
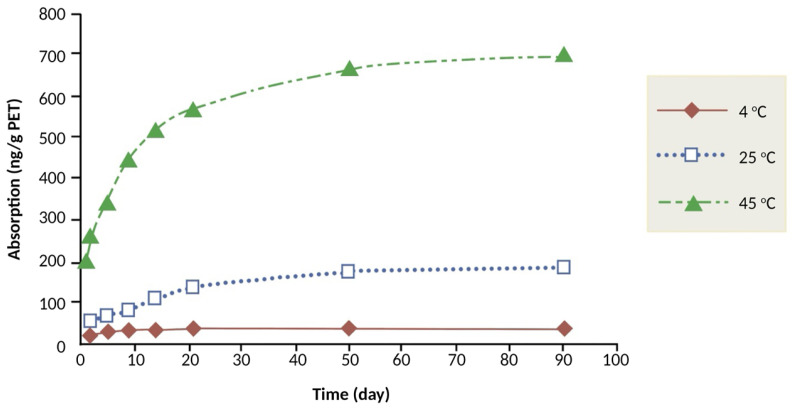
Absorption of menthol from yogurt drink into polyethylene terephthalate. (PET) bottles. From Farhoodi et al. [48] with permission.

Temperature is a key parameter regarding its effect on mass transfer processes. Mass transfer increases with increasing temperature according to the Arrhenius equation. Consequently, the sorption rate of flavors by polymers increases with temperature [9]. Tawfik et al. [46] as well as van Willige et al. [47] reported that temperature significantly influenced the absorption of flavor compounds by LDPE. Caner et al. [42] reported that temperature (40 °C and 60 °C) affected the degree of sorption of d-limonene by PP, PE/PA/EVOH/PE; and metPET/EVA/LLDPE. Higher temperature accelerates the sorption process due to greater molecular mobility and potential polymer swelling. Absorption of selected flavor compounds in a model solution by LLDPE, OPP, PC, PET film and bottle, and PEN was monitored over time at 4, 20, and 40 °C. Absorption by PC, PET, and PEN was much lower than by the polyolefins LLDPE and OPP. Temperature, however, did not affect the total amount of flavor absorption by LLDPE and OPP [2,23]. d-limonene absorption from citrus and cola beverages into PET bottles at three test temperatures (4, 25 and 40 °C) was investigated by Farhoodi et al. [9]. Results showed that flavor absorption increased with increasing temperature due to (i) increased mobility of the flavor compounds, and (ii) increased free volume of the polymer, resulting from the swelling of the polymer. The temperature effect on flavor compound sorption seems to be more important for PA, PET, metPET and PLA, compared to PE and PP which may be attributed to the Tg value of the glassy polymers being close to the storage temperatures. Tawfik et al. [46] demonstrated that PET stored for 15 d at 37 °C in a model solution containing 320 ppm limonene, absorbed seven times more limonene than when stored at 5 ◦C, but four times more after 45 d. Finally, Farhoodi et al. [48] reported that the amounts of menthol absorption into PET bottles increased with storage time and higher temperatures (Figure 3).

### 4.3. Food Composition

Food composition plays a key role in the extent of flavor compounds’ sorption by polymers. Besides the specific properties of the flavor compound and the polymer type, the possible interactions between the flavor and food components is very important (Naknean & Meenune) [49]. Flavor components may be absorbed, dissolved, chemically bound, mechanically entrapped or even encapsulated within the food matrix by food components. The extent to which each of these mechanisms dominates, depends on the properties of the flavor compound (molecular size, volatility, shape, functional groups, etc.) and the physical and chemical properties of the food components. Macromolecular constituents such as proteins and carbohydrates, as well as lipids interact with flavor compounds, resulting in change in the concentration of the available amount of the flavor compounds in solution which in turn affects their absorption [50]. Absorption of flavor compounds by LLDPE was studied by van Willige et al. [2] in different model systems representing different food matrix compositions. It was shown that the extent of flavor absorption by LLDPE followed the order: fat/oil > polysaccharides and proteins > disaccharides.

Lipids are present in a large variety of foods and may function as solvents for most flavor compounds; the lipophilicity of flavor compounds affects their retention within the fatty food matrix [50]. For example, lipids act as an aroma solvent reducing its interaction with PP (semi-crystalline polymer) and PS (amorphous polymer). Regarding the effect of packaging, PS containers are preferable for limiting the development of odor and aroma defects particularly in 4%-fat yogurts [51]. As many flavor compounds show a lipophilic character, food products with high fat/oil content show limited flavor absorption into LLDPE packaging material compared to foods with a low fat content [52]. van Willige et al. [2] investigated the absorption of limonene, decanal and linalool spiked in an LLDPE/milk (skimmed milk and whole milk) system. Limonene, decanal and linalool solubilized into the oily phase resulting in their reduced absorption by LLDPE. Oil affected the amount of flavor absorption by LLDPE much more than pectin (carbohydrate) or casein (protein). Oil-in-water (o/w) model emulsions spiked with ethyl esters, 2-nonanone, benzaldehyde were stored in PLA containers. PLA showed good compatibility with oil and the flavored oil-in-water emulsion [53].

Proteins, on their own, do not significantly contribute to flavor but affect flavor perception through the sorption and/or binding of flavor compounds. Aldehydes, for example, bind irreversibly, through covalent bonds to proteins as well as through hydrophobic interactions [54]. Factors that affect binding include: type of protein, protein conformation, concentration, temperature, and pH [55]. Li et al. [56] showed that vanillin had a higher affinity to whey proteins compared to soy and casein. It was also shown that proteins interacted irreversibly with decanal resulting in suppressed flavor absorption substantially by polymers [2,52]. Likewise, it was shown that b-lactoglobulin (b-lg) and casein were able to bind aldehydes, either temporarily or permanently, through hydrophobic or covalent interactions resulting in suppression of flavor absorption by LLDPE [2]. The binding of flavor compounds by proteins increases with increasing chain length (i.e., hydrophobicity) of the aroma compound [57].

Carbohydrates substantially affect aroma compound absorption. Between sugars and polysaccharides, the latter allow more chemical interactions due to the large variety of functional groups available [58] while the macromolecular chain of polysaccharides provides little opportunity for binding [59]. Binding mechanisms of flavor compounds include: adsorption, absorption, physical entrapment in micro regions, encapsulation and hydrogen bond formation [60].

The determination of partition coefficients of various flavor compounds by PE showed that sorption was greatly influenced by the presence of oil in solution, but not sucrose. For nonpolar compounds preferentially absorbed by PE, the addition of oil greatly suppressed flavor loss, the reduction being very significant in the presence of only 0.1% oil. The incorporation of flavors into oleoresins was suggested to develop beverages resistant to flavor scalping (available from: www.foodproductdesign.com, accessed on 1 April 2026)

According to van Willige et al. [2,52] the presence of carbohydrates [pectin and carboxymethyl cellulose (CMC)] suppressed the absorption of limonene and decanal from the food matrix into LLDPE. Polysaccharides, such as pectin and CMC, can increase viscosity due to their high molecular weight reducing the diffusion of flavor compounds from the food matrix into the polymeric film. Finally, sugars such as lactose and saccharose in a food matrix, may enhance the retention of more polar linalool and ethyl-2-methylbutyrate by LLDPE through water binding, causing a salting-out effect [28].

### 4.4. Relative Humidity

It has been documented that the presence of water vapor within a hydrophilic polymer enhances the diffusion of gases and vapors through the polymeric matrix. In this case, water diffusing through the polymeric packaging material acts as a plasticizer, enhancing mass transport. In recent decades, EVOH, a high-barrier material to organic vapors, has been used as a means to reduce food aroma scalping. However, in environments of intermediate or high water activity, the hydrophilic EVOH drastically loses its barrier properties as the solubility and diffusivity of flavor compounds increase dramatically, resulting in increased permeation of aromatic compounds [19]. Despite the above, EVOH outperforms LDPE as a barrier to organic vapors even at 100% RH [61].

## 5. Studies on Flavor Scalping in Selected Beverages and Foods

### 5.1. Flavor Scalping in Conventional Plastics

In a study by Charara et al. [13], citrus oil components which contribute substantially to flavor of orange juice, were shown to be absorbed into various polymeric materials [LDPE, HDPE, PP, and surlyn] used in aseptic packaging. Absorption was dependent on the ratio amorphous/crystalline nature of the polymers. Amorphous LDPE and Surlyn of low crystallinity showed high absorption potential. In contrast, PP and HDPE, polymers of high crystallinity, showed low absorption potential. The degree of absorption was also dependent on the nature of the citrus oil constituents. Absorption followed the order: terpene constituents > sesquiterpenes > aldehydes. Terpene hydrocarbons, lipophilic in nature, easily diffused into the amorphous matrices of the polymers. It was also shown that highly crystalline polymers (HDPE and PP) swelled less than partly crystalline LDPE and Surlyn. A loss of ca. 70% was recorded for limonene from orange oil in contact with LDPE after 4 d, while a respective loss of ca. 30% was recorded from orange oil in contact with HDPE and PP. Berlinet et al. [62] studied the evolution of flavor compounds from orange juice packaged in glass, monolayer PET (PET 1), multilayer PET (PET 2) and plasma-treated PET (PET 3). Containers were stored at room temperature under artificial light. Volatile compounds in juice samples were determined after 0, 2, 3 and 5 months of storage. Results showed that at the end of storage, only 0.2 to 0.3% of limonene and β-myrcene in the juice were absorbed by the plastic bottles. The evolution of aroma compounds was strongly correlated to storage time but not to the type of packaging material. The results suggested that the losses of flavor compounds from orange juice could be attributed to the high acidity of the matrix, implying acid-catalyzed reactions. A study by van Willige et al. [63] on the flavor absorption of seven flavor compounds added to model solutions by LDPE, PC, and PET showed valencene to be almost completely absorbed by LDPE, followed to a lesser extent by decanal, hexyl acetate, octanol, and nonanone. Fewer and less of the flavor compounds were absorbed from the model solution by PC and PET. However, limonene was readily absorbed from orange juice by LDPE, while myrcene, valencene, pinene and decanal were absorbed in smaller amounts. In a similar study on flavor absorption, Ayhan et al. [64] investigated the retention of aldehydes and ethyl butanoate in packaged orange juice. Results showed a significantly higher retention of flavor compound in the product packaged in PET compared to HDPE and LDPE. The presence of juice pulp in orange juice resulted in a decreased absorption of flavor compounds into polymeric containers. The authors postulated that pulp particles retain flavor compounds such as limonene, resulting in the decreased absorption of these compounds by the plastic containers. A review on packaging–flavor interactions by Linssen et al. [65] concluded that although such interactions occur, they do not critically affect food quality in commercial situations. The proof for this is that polyolefins in contact with juices are widely used commercially throughout the world. Imail et al. [66] investigated the sorption of selected flavor compounds of orange juice by polymeric food packaging materials. Three sealant films were tested: LDPE, EVOH and a co-polyester. Samples of the films were immersed in the juice for 24 days at 22 °C and the level of sorbed volatiles monitored as a function of time. Results showed that sorption of flavor compounds by the co-polyester film was significantly lower than the two others. Nielsen et al. [20] evaluated five polymer packaging films with respect to the sorption of ten apple aroma compounds from an aqueous solution. Results of the study showed major differences in the amount of aroma compounds absorbed by the different polymers. Nonpolar LDPE, LLDPE, and PP absorbed higher amounts of aroma compounds compared to more polar polymers. PP absorbed larger amounts of aroma compounds than the two polyethylenes, which was attributed to differences in crystallinity and morphology of the polymers. Likewise, Konczaland et al. [67] reported a higher sorption of apple aromas by LDPE compared to EVOH. Arora et al. [68] investigated the scalping of d-limonene, geranial, octanal, and decanal from juice products by the PE liner in brick type aseptic containers. As a result of flavor scalping, the packaged juice lacked in flavor notes characteristic of the fresh juice. PP ranked second after PE in the amount of flavor compound sorbed. According to reports, 62% and 37% sorption of nonpolar and polar flavor compounds occurs, respectively by PP. To tackle this problem, coextruded PP containers, consisting of PP outer and inner layers sandwiching an internal EVOH layer have shown a substantial decrease in d-limonene content compared to juices packaged in conventional laminated containers. Alternatively, a layer of Saran laminated to PP or HDPE is recommended to substantially reduce the sorption of d-limonene. Sheung et al. [69] developed a dynamic headspace gas chromatographic method (DH-GC) to study the absorption of d-limonene, α-pinene, ethyl butyrate, and octanal by laminated polymeric packaging materials including LDPE, PET, PVDC and EVOH copolymer. A test cell was designed to study orange juice flavor absorption into the packaging materials for 28 d at 25 °C. d-limonene and α-pinene were more absorbed by LDPE and EVOH than other packaging materials because of the nonpolar nature of the flavor compounds and that of the packaging materials. Ethyl butyrate or octanal absorption did not differ among the four packaging materials. Addition of a PET and PVDC layer to the LDPE packaging materials reduced the d-limonene and α-pinene absorption by 20% and 50%, respectively. Sadler & Braddock [70] studied the absorption of citrus flavor by LDPE. Limonene, ethyl butyrate, myrcene, and α-pinene were easily absorbed by LDPE while octanal, citral, linalool, and terpineol were absorbed at much lower amounts. Diffusion coefficients of flavor compounds in the polymer were calculated and found proportional to the flavor compounds’ solubility in the polymer. Bai et al. [71] investigated the interaction of green tea volatiles with different plastic packaging material during storage. Green tea staleness was associated with increased concentrations of alcohols, aldehydes, and ketones, the severity of which varied with the packaging material. PP performed better with respect to flavor scalping compared to PE and PET, with the aluminum–plastic composite packaging (AP) exhibiting the best performance, contributing to the stability of the product flavor during storage. Adams et al. [72] investigated the potential loss of product quality and potential damage to the package as the result of the sorption of food aroma compounds by packaging materials. The flavor scalping performance of 14 different LLDPE sealant resins was comparatively evaluated in stand-up pouch applications. Νo significant flavor scalping was observed in sunflower oil. A clear indication of flavor scalping were observed for limonene, decanal and 2(E)-nonenal in aqueous model systems. The extent of scalping for a given flavor compound was shown to be inversely proportional to its water solubility. A significantly higher scalping was recorded for polymeric sealants with the lowest density in contact with aqueous media. Despite the above, differences between the evaluated sealant resins were minor, and are not expected to affect sensory properties of the contained product in the sealant resins’ density range (0.902–0.919 g/mL) evaluated. You & O’Keefe [73] investigated the scalping potential of three different can linings made of polyolefin, acrylic, and epoxy resins for the binding of octanal, nonanal, decanal, eugenol and d-limonene. The experiment was carried out at room temperature over a 2-week period. Flavor compounds were studied at the concentration range 4–1000 ppb. Almost complete binding of all five flavor compounds was observed between days 9–14 days for each of the can linings. Yuan et al. [74] studied the flavor scalping potential of PET-lined steel material (PET-LS). Six scalping flavor compounds of PET-LS were determined: 2-pentanone, 2-heptanone, hexanal, citral, *n*-butyl acetate and isopentyl acetate. Storage temperature and initial compound concentration significantly affected sorption and diffusion of the aroma compounds. Both phenomena increased proportionally with increasing temperature and concentration (below 500 μL/L) with a sharp increase above this value. The Fickian diffusion model was used to fit the experimental kinetics data. Diffusion coefficient values were about the order of 10^−11^ to 10^−12^ m^2^/day, lower than those reported for other polymeric materials. The study suggested that the Fickian diffusion model could be used for the reliable prediction of absorption behavior for flavor compounds by PET-LS. Farhoodi et al. [48] studied the potential interaction of menthol with PET bottles at 4, 25, and 45 °C during a three month period. Such an interaction may affect the flavor intensity of the packaged product (i.e., yogurt drink). The absorbed flavor compounds were extracted from PET bottles at specific time periods and quantified. The diffusion coefficient of menthol into PET bottles was then determined After 90 days, the absorption amounts were 38.21, 186.66 and 700.50 ng/g of PET bottle, at 4, 25, and 45 °C respectively. The amounts of menthol absorbed by PET bottles increased with storage time and higher temperature. A significant increase in diffusion coefficient of menthol in PET bottle was noted with increased storage temperatures. Finally, Hosono et al. [75] investigated the use of the purge and trap (P&T) as well as solvent extraction methods to measure flavor sorption by plastic packaging materials. Twenty-one representative flavor compounds were sorbed from model flavor aqueous solutions by PET, LDPE, CPP films and multilayer pouches. The sorption values obtained from the P&T method were higher than those obtained using solvent extraction for all samples, and a high correlation was observed between the respective measured values of the two methods. The sorption of flavor compounds by the LLDPE and CPP films in the rubbery state at 23 °C was much higher than that on the PET films in the glass state. Furthermore, the CPP films showed higher sorption than the LLDPE films. Polyolefins exhibited higher sorption of compounds with longer carbon chains, while PET exhibited higher sorption of aromatic aldehydes. Furthermore, the multilayered pouches with a CPP film on the inner surface exhibited lower sorption compared to CPP films alone. This phenomenon may be attributed to the type of contact with the model aqueous solution; that is, the multilayered pouches were in one-side contact whereas the CPP films were in two-side contact with the model solution.

### 5.2. Flavor Scalping in Multilayer, Biodegradable and Recycled Packaging Materials

#### 5.2.1. Flavor Scalping in Multilayer Packaging Materials

Lee et al. [76] determined the absorption parameters of d-limonene through a multilayered film (high impact polystyrene (HIPS)/PVDC/LDPE). Orange juice was placed in a cell kept at room temperature in contact with the film for 72 h. The diffusion coefficient and the partition coefficient of d-limonene in the packaging film were approximately 1–2 × 10^−12^ m^2^/s and 0.03, respectively. These absorption parameters enabled the prediction of the absorption behavior of d-limonene by the multilayer film. In contrast to the above, Pieper et al. [77] reported no perceived sensory differences between orange juice packaged in glass containers and in laminated aseptic cartons despite the fact that absorption of up to 50% limonene and other hydrocarbons, small quantities of ketones occurred in the latter container at 4 °C. In a study by Schroeder et al. [78] two polyester/polyolefin laminated films coated with a PVDC copolymer were exposed to lemon juice, a hot sauce product and a food simulant at 45 °C. Sorption of flavor volatiles resulted in delamination of both laminates following exposure to the food products. d-limonene was identified as the primary component sorbed by the laminates. Commercially, most aseptically filled juices are packaged in LDPE-laminated cartons such as Tetra Brik and Combibloc. To tackle the flavor scalping problem, food industries often add excess flavors to the food for maintaining acceptable taste and flavor for consumers until the end of the product’s shelf life. Hwang et al. [79] prepared five multilayer packaging films: APET/PE, APET/PP, APET/PE with a UV inhibitor, APET/PP/PE, and APET/Barex/PP for blister packaging applications. Package/product compatibility with simulants (soy sauce and sunscreen skin cream) was evaluated at 37.8 C after 3, 7, 14, and 28 days. After exposure of the films to soy sauce, the APET/PE and APET/PP/PE films showed significant changes in color whereas APET/PP and APET/Barex/PP showed no differences. Color changes were attributed to the sorption of chemical compounds that caused the samples to appear more yellow. After exposure to the skin cream, APET/PE and APET/Barex/PP showed no significant color changes. APET/PP/PE and APET/PP showed statistically significant differences in DE* color value over time; however, these changes were not perceived with the naked eye.

#### 5.2.2. Flavor Scalping in Biodegradable Packaging Materials

Although the utilization of biodegradable polymers as packaging materials can greatly contribute to the sustainability of the packaging industry, only limited data on their aroma scalping properties are available. Mauricio-Iglesias et al. [80] studied the effect of high pressure thermal (HP/T) treatment on the absorption of 2-hexanone, ethyl butanoate, ethyl hexanoate, d-limonene by LDPE and PLA. Pasteurization (800 MPa, 40 °C) and sterilization (800 MPa, 115 °C) treatments were carried out on film samples in contact with four different food simulants spiked with flavor compounds. Results showed that the flavor compounds tested were found quite stable after the HP/T pasteurization in contrast to significant losses in flavor compounds after HP/T sterilization. With regard to flavor scalping, both materials proved suitable for HP/T pasteurization. On the contrary, LDPE melted during the conventional sterilization. With regard to flavor scalping by PLA, temperature proved to be of critical importance, especially if the treatment temperature exceeded the Tg of the polymer. According to Salazar et al. [53] the main component of most foods is water with PLA possessing a high affinity for water. Thus, the role of water in the transfer of aroma compounds through PLA should be of interest because water can affect the interactions between aroma compounds and the PLA packaging. Cihal et al. [81] evaluated the aroma scalping characteristics of a series of films made from biodegradable polybutylene succinate (PBS) and polybutylene succinate-co-adipate (PBSA) for common flavor compounds in foods (ethyl butyrate, ethyl exanoate, hexan-1-ol, heptanal and (*R*)-(+)-limonene). The aroma scalping of LDPE films was also evaluated for comparison purposes. Diffusion and permeability coefficients of the tested aroma compounds were determined at 23 °C. The permeability coefficients for the films made from PBS, PBSA and LDPE were comparable for the more polar compounds: hexan-1-ol, ethyl acetate and ethyl butyrate. In contrast, the permeability coefficients of the less polar aroma compounds (ethyl hexanoate, heptanal and (*R*)-(+)-limonene) in PBS- and PBSA-based films were by one to two orders of magnitude lower than those of the LDPE. Finally, Leelaphiwat et al. [82] determined the permeability (P), diffusion (D) and solubility (S) coefficients for eucalyptol, estragole, linalool and citral through commercial polymers (PLA, LDPE, PP, PA, PET and metPET. Results showed that *P* and *D* for all four aroma compounds were highest in LDPE, except for eucalyptol, in which *P* was slightly higher in PLA. The solubility coefficients were the highest in PLA, suggesting the highest affinity of PLA to these aroma compounds. The study concluded that *P*, *D* and *S* data of the four aroma compounds through the tested polymers can be useful in selecting the proper packaging material for preserving these specific aroma compounds in food products.

#### 5.2.3. Flavor Scalping in Recycled Packaging Materials

Flavor scalping is usually intensified in recycled plastics due to porous, partly degraded polymers and trapped contaminants from previous use or cross contamination due to mixing of waste materials, i.e., detergent/lubricant containers, etc. [83]. The scalping behavior is heavily influenced by the type of plastic, with nonpolar materials like PE and PP being most prone to absorbing lipophilic compounds. While high-quality recycled PET (rPET) can behave similarly to virgin material, it can still show slight changes in aromatic profiles of foods. Furthermore, the reuse of plastic packages has the potential to increase the level of chemical contamination, microbial film colonization on plastics’ surfaces and microplastic particles in foods that could reduce product hygienic and sensory food quality [84]. In such cases, it often causes significant off-flavors (e.g., leather, cardboard) as well as potential migration of contaminants.

Traditional washing of LDPE or PET with NaOH solutions can remove less than half of the sorbed flavor compounds, particularly terpenes. This means that contaminants from the original use can persist, creating off-flavor concerns in the recycled product. Triantafyllou et al. [85] extracted various recycled PET bottles with either CHCl3, CH2Cl2 or a mixture of the two solvents and attempted to identify and quantify sorbed compounds. No measurable amounts of contaminants (either specific recycling compounds or compounds coming from the prior use of PET) were identified in most cases. In specific PET samples only compounds originating from the first use of the PET bottle, i.e., limonene and traces of other soft drink flavorings like γ-terpinene and p-cymene were detected and quantified in low ppm amounts. Finally, Cabanes et al. [83] reviewed the literature regarding all the organic compounds (VOCs) and odor-active substances identified in virgin and recycled polymers, aiming to evaluate whether there is a notable difference between them based on the chemical structure of the emitted VOCs. The study concluded that a large number of volatile organic compounds, especially flavor and fragrances-based substances as well as oxygenates, appear in post-consumer plastic waste compared to virgin polymers.

### 5.3. Adhesives and Inks as a Source of Off-Flavors

Common packaging materials for snacks such as potato chips, flavored puffed cereals, etc., are multilayer laminated films usually produced by combining numerous monolayer films, i.e., polyamide/polyethylene (PA/PE) or metallized polyester/polyethylene (metPET/PE) films. In one case, a commercial flavored puffed cereal snack developed a strong “solventlike,”objectionable odor. The source of the problem was traced to the residual solvent (ethyl acetate) used as the solvent in the adhesive used to bind the PA layer to the PE layer. The residual solvent in the laminated packaging film was the result of inadequate drying of laminate after the adhesion step [86]. In another case, a manufacturer of nondairy coffee creamers observed that shipping boxes of his foil-wrapped product had a severe musty odor. Investigation showed that all of the new production samples had the same odor problem identified as 1,3,5-trimethylbenzene. The compound corresponding to the trimethylbenzene peak had a strong musty odor identical to the odor of contaminated boxes. Further investigation revealed that the packaging supplier was using a new type of ink containing high levels of 1,3,5-trimethylbenzene for graphics on the foil packets [7]. Even though both above case studies comprise illustrative industrial observations they are characteristic of problems caused by flavor scalping. In a similar study, inks and model ink components were deliberately impregnated into carton boards at low temperature. During microwave heating, benzophenone was shown to migrate from the PE-coated boards to the packaged food. The phenomenon was attributed to the permeability of PE to low MW substances [87]. Migration of benzophenone, benzylbutyl phthalate, butyl benzoate, chlorodecane, and dimethyl phthalate was also detected after storing the food at −20 °C for 1 week in the impregnated carton board. It was concluded that, for inks used to print food contact materials, the content of low MW volatiles should be controlled to lower the migration levels potentially affecting the food sensory properties. In yet another study, an off-flavor in fruit flavored soft drinks packaged in flexible pouches made of a polyester/aluminum foil/polyethylene laminate resulted from foil/polyethylene laminate improper drying of the adhesive used in the lamination process. The off-flavor was identified as toluene in the faulty pouches and quantified at concentrations 26–68 times greater than that in the good pouches [88]. Finally, an off-odor with a smell of cat urine was reported in cooked ham products packaged in polyamide/surlyn laminate films [89]. Investigation of the problem identified the presence of diacetone alcohol (DAA), used in the printing ink of the film that eventually migrated into the laminate. Dehydration reaction of DAA by the surlyn packaging film resulted in the formation of 4-methyl-4-mercapto-pentane-2-one a compound with a distinct off-odor identical to the initial off-odor noted. It was proposed that when printing surlyn films or their laminates the use of diacetone and acetone should be avoided. Other factors that may affect package flavor contribution during film conversion include processing temperature, sealing temperature, sterilization, residual solvent from printing inks such as ethyl acetate, hexane, pentanol, toluene and heptane types of adhesives, coatings and extrusion processes [90].

Finally, another example of a type of product that developed styrene taint problems is that of coffee creamers and condensed milk packaged in single-serve thermoformed PS cups [91]. A survey of 22 coffee creamer packs carried out by the British Ministry of Agriculture Foods and Fisheries (MAFF) [92] reported styrene monomer levels in the product in the range from 23 to 223 μg/kg. The highest styrene content in the product was found in the mono-material PS with lower styrene values being recorded in the PS/PE laminate. The lowest styrene migration values were recorded in the PS/EVOH/PE. Despite the fact that the PS/EVOH/PE is a high-barrier material, the product packaged in this multilayer material still contained a perceptibly detectable amount of styrene at the end of its shelf life. The possible explanation for styrene in the product has to do with the fact that styrene from the PS layer transferred to the inner PE layer during shipment and storage. This took only a few days due to the relatively high diffusion coefficient of styrene in PS and PE. This also occurred in the PS/EVOH/PE high-barrier material but to a lesser extent, due to the EVOH barrier, and over a longer time period.

The migration of styrene from PS cups into different aqueous (milk of varying fat content, cold and hot beverages, soup, yogurt, ice cream) and fatty foods (olive oil) as well as in food simulant systems (3% acetic acid, 15%, 50%, or 100% ethanol) was found to be dependent on product fat content, with the maximum migration occurring in fatty foods [93]. More specifically, styrene migration from PS foam articles showed that the amount of styrene migrated into food oil was proportional to the square root of the time of exposure [94].

It should be clarified that the content of the present section lies in the interface between migration and flavor scalping based on the definition of the latter [Section 1(C)]. In the case studies reported in this section, migration directly affected the packaged food sensory properties.

### 5.4. Flavor Scalping in Wine

**Figure 4:**
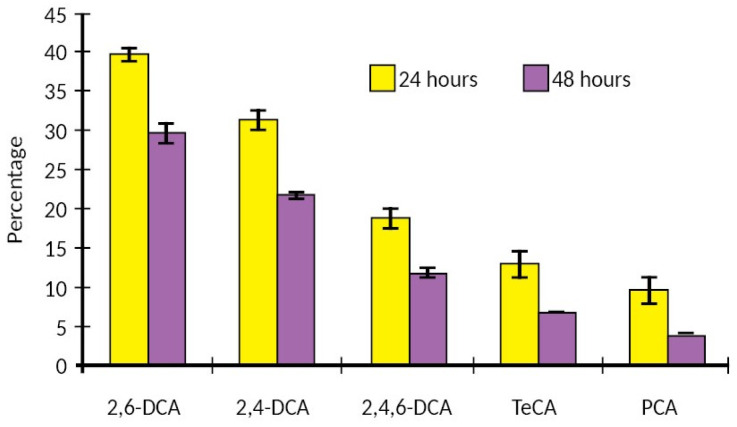
Absorption of chloroanisols after 48 h from wine into bottle closures. 2,6-DCA = 2,6-dichloroanisole, 2,4-DCA = 2,4-dichloroanisole, 2,4,6-TCA = 2,4,6-trichloroanisole, TeCA = 2,3,4,6-tetrachloroanisole, PCA = pentachloroanisole. From Capone et al. [95] with permission.

**Figure 5:**
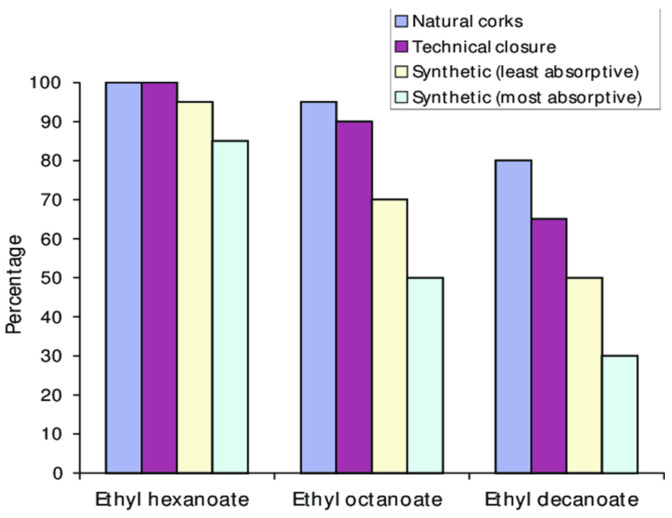
Absorption of longer chain esters from white wine into bottle closures after 2 years. From Capone et al. [95] with permission.

Flavor scalping often occurs in bottled wine. In the case of glass packaging, due to the inert nature of glass, the primary cause of flavor scalping known as ‘cork taint’ is the closure. Considerable research work on wine has been carried out with regard to cork taint [95,96,97,98]. The main compounds responsible for cork taint are the anisole derivatives: 2,4,6-trichloroanisole (TCA), 2,3,4,6-tetrachloroanisole (TECA), and pentachloroanisole (PCA). Of these, 2,4,6-TCA and 2,3,4,6-tetrachlorophenol have extremely low-flavor thresholds, contributing a moldy odor (or moldy cork) [97]. Thus, use of compounds containing chlorine during the steps of the manufacture of cork introduces the risk of imparting an off-flavor. Capone et al. [95] investigated the evolution of volatiles in white wine packaged in glass bottles sealed with natural corks. The long-term study at room temperature lasted two years (study 1) while the short-term study at 28 °C lasted for two weeks (study 2). The second study was run as there were indications that absorption of wine volatiles occurs in very short time periods. Figure 4 shows the absorption of chloroanisoles in wine after 24 and 48 h at 28 °C (short-term study). The long-term study concluded that (i) wine extracted small amounts of TCA from the cork closure and (ii) corks also absorbed substantially higher amounts of TCA and other chloroanisoles from wine. In the long-term study, various types of closures were used (glass ampule-no closure, metal screw cap, natural cork, agglomerated/technical cork and synthetic cork). In both studies, oak-related compounds as well as short-chain esters in the wine showed a negligible decrease over time, unaffected by closure type. In contrast, longer chain esters were readily absorbed at varying levels by different closures. Natural corks showed the lowest absorption while synthetic closures showed the highest absorption. Specifically, the absorption of ethyl decanoate after two years of storage was approximately 20, 30, 50 and 70% of that for the natural cork, agglomerated cork and synthetic cork (less absorptive and most absorptive) respectively (Figure 5). Negligible flavor scalping of longer chain esters was shown by glass ampules and metal screw caps.

Liu et al. [99] studied the effect of natural cork, agglomerated cork, and three types of synthetic closures on the flavor profile and sensory attributes of Chardonnay wine after 48 months of storage. Statistically significant differences in flavor absorption were recorded among the various bottle closures for the following volatile compounds: acetoin, 1-butanol, 2-phenylethanol, 1-pentanol, (Z)-3-hexen-1-ol, 2-nonanol, and ethyl decanoate. Sensory analysis unveiled that cork closures, both natural and technical, and the two synthetic closures with the lowest oxygen transmission rates (OTRs) preserved more fruity and flowery attributes, while the synthetic closures with the highest OTRs contributed more ‘grilled’ tones to the wines. According to Blake et al. [100] the absorption of flavor compound in wines by cork stoppers can have either a negative or positive impact on wine flavor. Volatile phenolic compounds, such as guaiacol, 4-methylguaiacol, 4-ethylguaiacol, 4-propylguaiacol, 4-vinylguaiacol, 4-ethylphenol, and eugenol, which are commonly present in wine, contribute negatively to wine flavor and can be absorbed on the cork surface [101]. The absorption phenomenon is more evident in synthetic closures compared to natural corks and has not been reported for metal screw caps.

Naphthalene has also been reported to be absorbed by natural and technical corks. 1,1,6-trimethyl-1,2-dihydronaphthalene (TDN) was described as the most strongly absorbed by natural and technical cork stoppers [95]. This molecule, although unpleasant in other wine cultivars, is characteristic of Riesling aged white wines, conferring a particular kerosene flavor [102]. Finally, natural and agglomerated cork stoppers have shown considerable sorptive capacity for methoxypyrazines [103]. These compounds are potent odor-active constituents of wine and are responsible for masking the fruity and floral aromas while generating undesired attributes similar to green bell pepper and vegetables.

Synthetic closures have shown a much higher capacity to absorb nonpolar compounds compared to cork stoppers as synthetic closures are made of LDPE, resulting in the development of unbalanced wine flavor characteristics [7]. According to Capone et al. [104] PE film in contact with wine for only four days resulted in decreased floral and fruity aromas of the wine. Overall, studies clearly show that the use of synthetic closures in bottled wine compared to natural cork is detrimental to wine quality due to the scalping of numerous volatile compounds, such as esters and organic acids [95]. Finally, De La Burgade et al. [105] studied the impact of wine closure permeability on flavor scalping. For this purpose, the absorption of volatile sulfur compounds (VSCs) on four micro-agglomerated wine cork closures was investigated by soaking them in model solutions and Shiraz wines for 7 days. Results showed that kinetically, most of the VSCs were quickly sorbed/scalped after only 1 h of soaking, with scalping increasing and reaching a plateau after 6 h. With regard to the amounts sorbed, VSC sorption on closures were 1% to 5% of the initial amount of VSCs present in the wine, suggesting a negligible impact on wine sensory properties.

**Figure 6:**
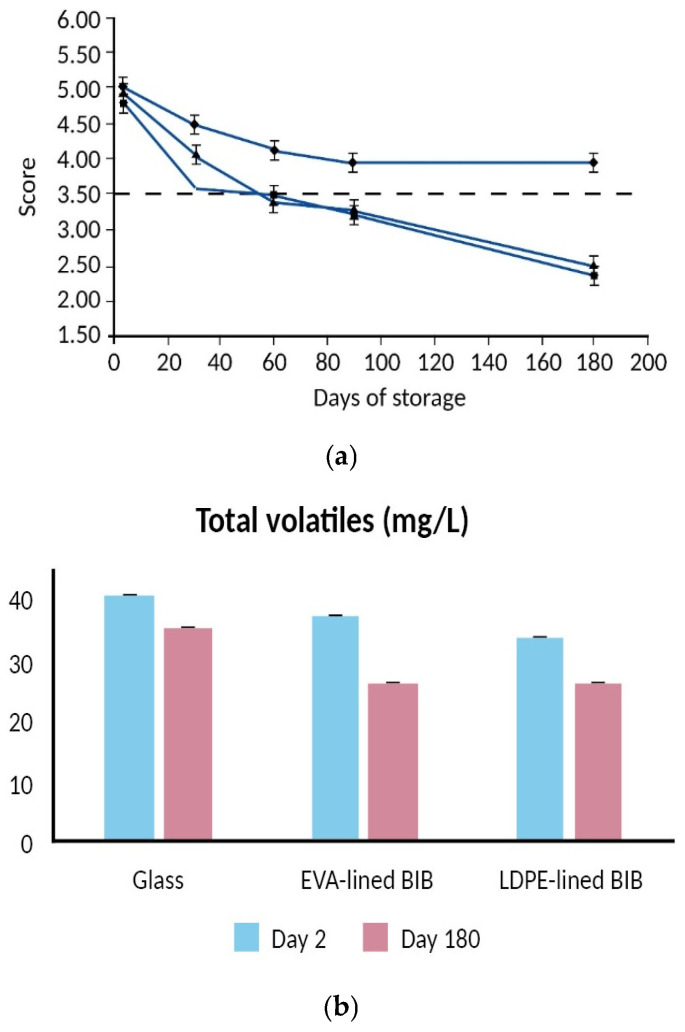
(**a**) Sensory evaluation (acceptability test) of Vilana white wine packaged in glass. (♦), in BIB lined with EVA (▲) and in BIB lined with LDPE (■) at 20 °C. Score is the mean of individual scores for appearance, odor and taste. Dash line indicates the lower limit of product acceptability. (**b**) Changes in total volatile compounds in Kotsifali red wine as a function of packaging material (glass and BIB) and storage time at 20 °C, respectively from Revi et al. [106] and Kosmadaki et al. [107].

With regard to the packaging of wine in plastics containers, Revi et al. [106] packaged white wine in (i) dark colored glass, (ii) an LDPE-lined bag-in-box (BIB) pouch and (iii) an EVA-lined (BIB) pouch. Packaged wines were stored for 6 months at 20 °C. Results showed that a substantial amount of the wine volatile compounds was absorbed by the plastic materials or lost to the environment through leakage of the valve fitment. The LDPE-lined pouch showed a considerably higher flavor scalping compared to the EVA-lined pouch. Wine packaged in glass showed negligible sorption of wine volatile compounds. Sensory evaluation showed that white wine packaged in glass bottles was of acceptable quality for at least 6 months vs. only 3 months for wine packaged in both plastic pouches (Figure 6a).

In a similar study, Kosmadaki et al. [107] packaged red wine in the same as above packaging materials and reported a significant sorption of the wine volatiles by the plastics BIB pouch materials as early as day 2 of storage (Figure 6b). Wine packaged in glass showed the best performance with respect to the retention of its aroma compounds during a 6-month storage period. Sensory evaluation showed that red wine packaged in glass bottles was judged of acceptable quality for 60 days vs. 30 days for the wine packaged in both BIB pouches. Finally, Moreira et al. [108] investigated the effect of different packaging configurations on the volatile composition and sensory properties of a white wine. BIB bags and glass bottles sealed with two different cork stoppers, natural and synthetic, were used in the experiments. Wine sampling was carried out before packaging and after 3, 6 and 12 months of storage. Results showed that wines packaged in BIB presented a significantly lower amount of selected volatile aroma compounds compared to glass bottles after 6 months of storage.

## 6. Legislation

Βesides the major concern in packaging regarding the potential migration of low MW compounds from the packaging material into the food product, posing food quality and safety problems, an additional concern is whether mass transfer between the food and the packaging material will affect sensory attributes (appearance, color, texture, odor and taste) of the contained product, all characteristics that may affect consumer acceptance.

The European Food Safety Authority (EFSA) regulates flavorings through EU Commission Regulation 1334/2008 [6], ensuring that they do not pose safety risks, including issues related to migration from packaging. While no specific regulation exists solely for “flavor scalping,” it is controlled under general safety requirements for food contact materials and the requirement that flavoring substances must be safe for human consumption. EFSA regulates Food Contact Materials (FCMs) under EU Framework EU Commission Regulation 2004/1935 [109], ensuring that materials do not transfer constituents to food that endanger health, alter food composition, or impair organoleptic characteristics. In the USA, FDA regulations also do not specifically refer to “flavor scalping,” but control this phenomenon via strict food contact substance (FCS) regulations under 21 CFR Parts 174–186 [110], requiring that packaging materials do not transfer substances to food in harmful amounts. Under FSMA (21 CFR Part 117) [111], manufacturers must conduct hazard analyses to identify and mitigate risks, including chemical migration or absorption.

On 16 March 2025, EU Commission Regulation 2025/351 [112] entered into force in the European Union. This regulation introduces significant amendments to EU Commission Regulation 10/2011 [113] on plastic food contact materials (FCMs) and articles (FCAs), as well as minor updates to EU Commission Regulation (EU) 2022/1616 [40] on recycled plastics FCMs and EU Commission Regulation 2023/2006 [114] on good manufacturing practice of FCMs and FCAs. Some of the topics addressed in the regulation include the scope of existing legislation, purity standards to address non-intentionally added substances (NIAS), as well as labeling and testing requirements for repeat use articles. More specifically, with regard to recycled plastics materials, EU Commission Regulation 2022/1616 [40] on recycled plastic materials and articles sets out a legal framework to implement the above approach. The Regulation emphasizes two key concepts providing definitions for (1) Recycling Technology, Process, and Installation and (2) Pre-processing, decontamination, and post-processing of plastic waste.

A packaging material of paramount importance, with regard to flavor scalping, for packaged foods is PE (LDPE, LLDPE, HDPE). Depending on the source and processing of PE when in contact with aqueous foods, it can produce characteristic off-flavors in addition to the sorption of food flavor compounds. The description of such off-odors range from candle-like, stuffy, musty and rancid. For this reason, PE used for food packaging applications has specific high-quality standard requirements. Another commonly used packaging material, for which quality and safety have been extensively studied is polystyrene, due to the toxicity of its monomer, styrene. Besides its toxicity, styrene is known to impart a characteristic, detectable “plastic” or chemical taste to dairy products, particularly when migration levels are high. Extensive studies on the subject reported no health or quality consequence at the levels commonly found in foods. As of 2025, the European Union is implementing a specific migration limit (SML) for styrene in food contact materials (FCMs) of 40 μg/kg (40 ppb) following a 2024/2025 re-assessment by EFSA. This limit was established under [114], particularly addressing safety concerns for food packaged in polystyrene. Other packaging materials such as polyesters absorb very low amounts of flavor compounds, which do not influence product sensory quality.

## 7. Analytical Techniques to Determine Flavor Sorption

Numerous methods have been employed to determine flavor compounds involved in flavor scalping. Among them solvent extraction, purge and trap (P&T) and solid phase microextraction (SPME) are the most widely used today [75]. Among them solvent extraction requires laborious extraction of volatile compounds from the packaging material using flammable organic solvents and solvent concentration steps before analysis. P&T is a dynamic headspace method in which volatile compounds are collected in the vapor phase from an aqueous sample via purging with an inert gas (i.e.,N_2_) and then trapped in a trap tube. After collection, the trap tube is heated to desorb the compounds, which are then transferred to the injection port of a gas chromatograph (GC) or gas chromatograph–mass spectrometer (GC–MS) for analysis. The P&T method does not require solvents or concentration steps. SPME is based on the partitioning of analytes (i.e., flavor compounds) between a sample matrix (liquid or gas) and a stationary phase (a thin polymeric coating) immobilized on a fused silica fiber. Upon exposure of the fused silica fiber to the headspace of a vial containing the polymer sample, flavor compounds are sorbed on the polymeric coating, the silica fiber is retracted and inserted into the GC injection port for analysis. SPME is a solvent-free sample preparation technique that integrates sampling, extraction, concentration, and sample introduction into a single step.

Alternative extraction methods for measuring flavor sorption have been reported in the literature, including multiple headspace techniques, extraction using supercritical fluids, solid phase extraction and thermal desorption methods [75]. A method combining HS-SPME with two-dimensional gas chromatography and quadrupole time-of-flight mass spectrometry (QTOF-MS) (HS-SPME-GC × GC-QTOF-MS) has also been developed for the analysis of volatile organic compounds (VOCs) in food contact paperboard [115].

## 8. Conclusions—Future Perspectives

Based on the available literature, there is sufficient evidence that sorption of flavor constituents into the packaging material, and conversely, migration of odorous molecules from the packaging material to the food results in the overall flavor deterioration of packaged foods and beverages. Furthermore, flavor scalping, besides affecting the sensory properties of the contained food, can also affect the performance of the plastics material itself, by (i) enhancement of the permeability to flavor compounds and oxygen due to polymer swelling and (ii) delamination of multilayer structures causing package failure. Understanding the potential interaction of a particular food/beverage of characteristic flavor with a given polymer can lead to a more efficient package design resulting in the retention of optimum quality of the packaged product as well as the optimal performance of the packaging materials. This comprises the challenge of scientists such food flavorists, analytical chemists and packaging specialists, who must work closely together to achieve the above goal. As new high-barrier packaging materials, such as PEN or multilayer plastics based on EVOH or polyvinylidene chloride (PVDC), are developed and new applications are adopted for existing materials, adequate functional barriers to odor transfer to and from the packaging material will be achieved.

Key trends and challenges shaping the future of flavor scalping may include:

(1) Packaging solutions including active and intelligent packaging focusing on material science, such as advanced monolayer and multilayer polymer films, that provide higher barriers to prevent absorption and chemical migration.
(2) Stricter regulations, on the part of EFSA and the FDA’s enforcing a reduction in chemical migration from packaging into food, along with the need for alternatives to packaging materials prone to scalping.
(3) The drive toward the use of biodegradable and plant-based packaging materials with specific flavor retention characteristics.
(4) The use of natural flavors, inherently more complex and volatile than artificial alternatives, requiring the design of packaging materials with reduced interaction potential with the packaged product.

## Figures and Tables

**Table 1:** Approximate partition coefficient values (kp) for various plastic/food package systems.

Substance	Polymer (P)	Food Phase (L)	k_P_/L
Nonpolar (n-alkane)	Nonpolar (PO)	Very polar (water)	1 × 10^5^–1 × 10^6^ (40,000)
Nonpolar to middle polarity (d-limonene)	Nonpolar (PE)	Very polar (water)	1200
Nonpolar to middle polarity (d-limonene)	Nonpolar (PO)	Polar (fruit juices)	400–600
Middle polarity (ketone)	Middle polarity (PVC)	Polar (ethanol)	10–20
Middle polarity (ester)	Nonpolar (PO)	Very polar (water)	10–40
Nonpolar (large n-alkanes)	Nonpolar (PO)	Polar (ethanol)	5
Nonpolar (n-alkane)	Nonpolar (PO)	Nonpolar (n-alkane)	1
All polarities	All polymers	Oil	0.1–10
Polar (alcohol)	Very polar (PA)	Very polar	1
Polar to middle polarity (alcohol and H/C)	Polar (PET)	Polar	0.1–3
Polar (alcohol)	Nonpolar (PO)	Very polar (water)	0.5
Polar (alcohol)	Middle polarity (PVC)	Polar (ethanol)	0.1 (0.07)
Polar (alcohol and middle polarity)	Very polar, polar	Polar	>0.1
	(PA)	(propanol)	0.05–0.1
	(PET)	(methanol)	0.001–0.05
Very polar (acid)	Nonpolar (PO)	Very polar (water)	0.001 (0.001)

H/C = Hydrocarbon, PO = Polyolefin, PE = polyethylene, PVC = Polyvinyl chloride, PA = Polyamide, PET = polyethylene terephthalate. From Baner, 2000 [27] with permission.

## Data Availability

No new data were created in this study. The article reviews literature data. Data sharing is not applicable to this article.
